# Newcastle Disease Virus-Like Particles Displaying Prefusion-Stabilized SARS-CoV-2 Spikes Elicit Potent Neutralizing Responses

**DOI:** 10.3390/vaccines9020073

**Published:** 2021-01-21

**Authors:** Yongping Yang, Wei Shi, Olubukola M. Abiona, Alexandra Nazzari, Adam S. Olia, Li Ou, Emily Phung, Tyler Stephens, Yaroslav Tsybovsky, Raffaello Verardi, Shuishu Wang, Anne Werner, Christina Yap, David Ambrozak, Tatsiana Bylund, Tracy Liu, Richard Nguyen, Lingshu Wang, Baoshan Zhang, Tongqing Zhou, Gwo-Yu Chuang, Barney S. Graham, John R. Mascola, Kizzmekia S. Corbett, Peter D. Kwong

**Affiliations:** 1 Vaccine Research Center, National Institute of Allergy and Infectious Diseases, National Institutes of Health, Bethesda, MD 20892, USA; yongpiny@niaid.nih.gov (Y.Y.); shiw@mail.nih.gov (W.S.); olubukola.abiona@nih.gov (O.M.A.); alexandra.nazzari@nih.gov (A.N.); adam.olia@nih.gov (A.S.O.); li.ou@nih.gov (L.O.); emily.phung@nih.gov (E.P.); raffaello.verardi@nih.gov (R.V.); shuishu.wang@nih.gov (S.W.); anne.werner@nih.gov (A.W.); christina.yap@nih.gov (C.Y.); dambroza@mail.nih.gov (D.A.); tatsiana.bylund@nih.gov (T.B.); tracy.liu@nih.gov (T.L.); rnguyen@mail.nih.gov (R.N.); wangling@niaid.nih.gov (L.W.); baoshan.zhang@nih.gov (B.Z.); tzhou@mail.nih.gov (T.Z.); gwo-yu.chuang@nih.gov (G.-Y.C.); bgraham@mail.nih.gov (B.S.G.); jmascola@mail.nih.gov (J.R.M.); kizzmekia.corbett@nih.gov (K.S.C.); 2 Electron Microscopy Laboratory, Cancer Research Technology Program, Leidos Biomedical Research, Inc., Frederick National Laboratory for Cancer Research, Frederick, MD 21702, USA; tyler.stephens@nih.gov (T.S.); yaroslav.tsybovsky@nih.gov (Y.T.)

**Keywords:** neutralizing antibody, Newcastle disease virus, prefusion-stabilized spike, SARS-CoV-2, S-2P stabilization, vaccine, virus-like particle, VLP

## Abstract

The COVID-19 pandemic highlights an urgent need for vaccines that confer protection from SARS-CoV-2 infection. One approach to an effective COVID-19 vaccine may be through the display of SARS-CoV-2 spikes on the surface of virus-like particles, in a manner structurally mimicking spikes on a native virus. Here we report the development of Newcastle disease virus-like particles (NDVLPs) displaying the prefusion-stabilized SARS-CoV-2 spike ectodomain (S2P). Immunoassays with SARS-CoV-2-neutralizing antibodies revealed the antigenicity of S2P-NDVLP to be generally similar to that of soluble S2P, and negative-stain electron microscopy showed S2P on the NDVLP surface to be displayed with a morphology corresponding to its prefusion conformation. Mice immunized with S2P-NDVLP showed substantial neutralization titers (geometric mean ID_50_ = 386) two weeks after prime immunization, significantly higher than those elicited by a molar equivalent amount of soluble S2P (geometric mean ID_50_ = 17). Neutralizing titers at Week 5, two weeks after a boost immunization with S2P-NDVLP doses ranging from 2.0 to 250 μg, extended from 2125 to 4552, and these generally showed a higher ratio of neutralization versus ELISA than observed with soluble S2P. Overall, S2P-NDVLP appears to be a promising COVID-19 vaccine candidate capable of eliciting substantial neutralizing activity.

## 1. Introduction

The development of effective SARS-CoV-2 vaccines is a global health priority [1,2]. Although multiple vaccines are proceeding through clinical trials, some of which are achieving high levels of efficacy against COVID-19 [3,4,5,6], it seems prudent to explore the development of second-generation vaccines for improved immunogenicity, manufacturability, and scalability to fill gaps in the global vaccine portfolio. Particularly for immunogenicity, improvements could be made in the speed, potency, and durability of neutralizing antibody responses and/or T-cell responses [5,7,8,9,10,11,12,13,14,15]. The SARS-CoV-2 spike glycoprotein is the main target for neutralizing antibodies and has, therefore, been a focus of vaccine design efforts [16,17]. The spike is a homotrimer, with each spike protomer initially synthesized as a single polypeptide, which can be cleaved by furin into two subunits, S1 and S2 [18]. The membrane-distal S1 subunit contains the N-terminal domain (NTD), the receptor-binding domain (RBD), and two subdomains (SD1 and SD2). The S2 subunit contains the viral fusion machinery, including a fusion peptide, which is liberated by TMPRSS2 or the cathepsin cleavage, and HR1 and HR2 regions, which rearrange between prefusion and postfusion states to drive virion- and target cell-membrane fusion [19,20]. S2 is attached to the viral membrane through a transmembrane-spanning region, followed by a C-terminal tail inside the virion.

The majority of current SARS-CoV-2 vaccines and vaccine candidates in preclinical or clinical trials are based on the spike glycoprotein or the RBD. These use diverse vaccine delivery modalities, including mRNA, recombinant DNA, inactivated virus, and various viral vectors, and have been reported to elicit neutralizing antibodies, with primarily Th1-biased responses and up to 95% protection against symptomatic COVID-19 disease [3,5,14,15,16,21,22,23,24,25,26,27]. To design second-generation SARS-CoV-2 vaccines, current efforts have focused on nanoparticle-based, viral-vector-based, or virus-derived replicon RNA-based vaccine candidates and virus-like particle (VLP) versions of immunogens. Protein nanoparticles displaying spike or RBD have been shown to elicit greater than 10-fold higher neutralizing titers than non-nanoparticle immunogens [10,11]. Current vector-based viral particle platforms to present SARS-CoV-2 antigens include the Moloney murine leukemia virus (MLV)-vectored COVID-19 vaccine [28], Newcastle disease virus (NDV)-vectored COVID-19 vaccine [9], rabies-virus-based COVID-19 vaccine [7], vesicular stomatitis virus (VSV)-vectored COVID-19 vaccines [29,30], virus-derived replicon RNA-based COVID-19 vaccines [8,31], and recombinant adenovirus-vectored COVID-19 vaccines [5,32,33]. Viral vector or virus-derived replicon RNA-based vaccine candidates should be able to produce molecules antigenically equivalent to those displayed on SARS-CoV-2 virions and should thus be able to induce humoral immune responses that are similar to those observed in people with SARS-CoV-2 [8]. However, some viral vaccine candidates have been associated with biological safety concerns, including the potential for viral genome integration, as observed with human adenovirus- or measles virus-vectored vaccines, although these vaccines are considered quite safe [34,35].

VLPs are assemblies of viral structural proteins without genetic material. Because of their resemblance to a real virus, VLPs are preferentially recognized by antigen-presenting cells and are often capable of inducing high-level immune responses. VLP vaccines have been developed from human papillomavirus and hepatitis B virus [36]. There are also reports of using SARS-CoV-2 structural proteins, such as envelope, membrane, and nucleoprotein, to develop VLPs [37]. NDV is an avian pathogen, typically causing no symptoms in humans, although, in rare cases, mild symptoms have been reported. NDV structural proteins, fusion (F), nucleoprotein (NP), and matrix (M), together, can form a stable-membraned VLP (NDVLP) [38,39]. NDVLPs have been used to display other viral antigens, such as human respiratory syncytial virus (RSV) glycoproteins F and G, by replacing the NDV F ectodomain with RSV antigens [40]. As vectored antigens can present on the surface of NDVLP without other viral proteins (other antigenic determinants being hidden behind an encapsulating lipid membrane), NDVLP is expected to induce high-level immune responses.

In this study, we engineered a prefusion-stabilized version of the SARS-CoV-2 spike (S2P) by replacing its transmembrane and cytoplasmic regions with those from the NDV fusion protein to create a construct we named S2P-NDV-Ftmct. Coexpression of S2P-NDV-Ftmct with NDV matrix and nucleocapsid proteins in 293T cells led to the secretion of SARS-CoV-2 spike membrane-anchored NDVLP (S2P-NDVLP) into the cell culture medium. Purified S2P-NDVLPs were analyzed for their spike content, antigenicity, and immunogenicity in mice. Overall, S2P-NDVLP appears to be a promising COVID-19 vaccine candidate.

## 2. Materials and Methods

### 2.1. Cell Line

HEK293T cells (#CRL3216, ATCC, VA) or transfecting HEK293T cells were cultured in Dulbecco’s Modified Eagle’s Medium (DMEM, Thermo Fisher Scientific, Carlsbad, CA, USA) containing 10% inactivated fetal bovine serum (FBS, ABI Scientific, Chantilly, VA, USA), 2 mM glutamine, and 1× Streptomycin–Penicillin (Thermo Fisher Scientific) at 37 °C and 5% CO_2_.

### 2.2. Preparation of Plasmids

**Figure 1:**
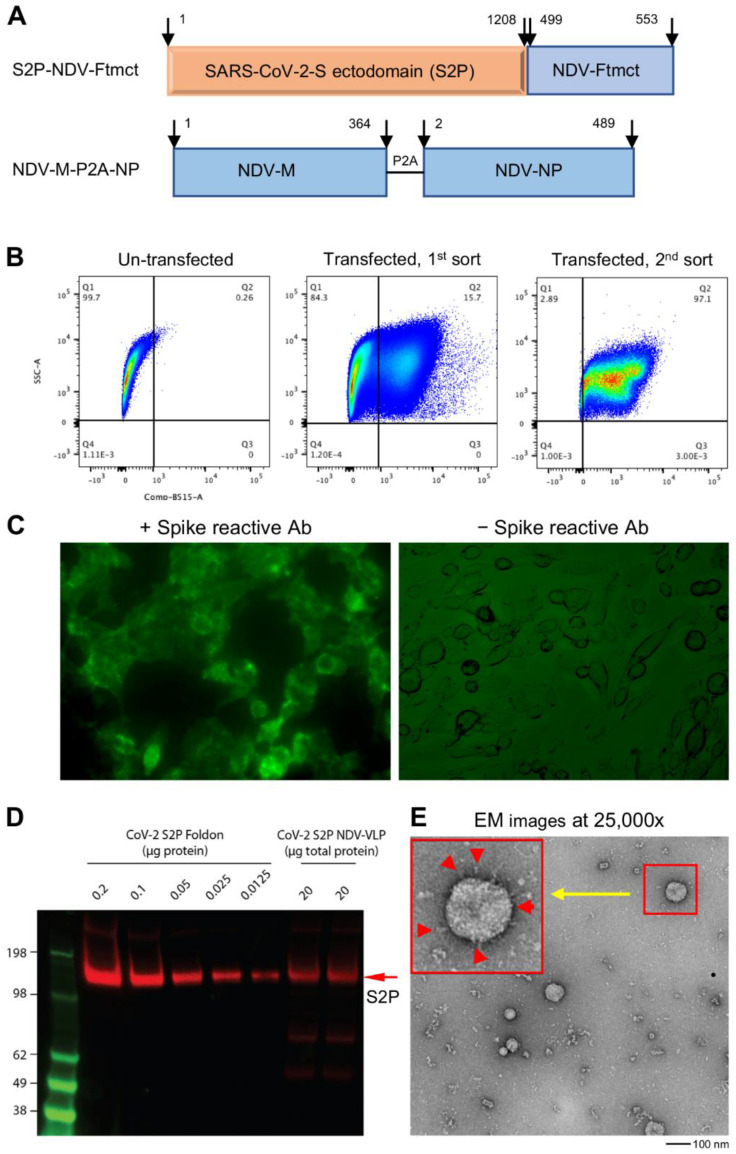
Generation and characterization of SARS-CoV-2 spike membrane-anchored on Newcastle disease virus-like particle (S2P-NDVLP). (**A**) Schematic construction of S2P-NDVLP-expression plasmids. The top linear diagram shows a S2P-NDV-Ftmct chimeric construct, with the SARS-CoV-2 prefusion-stabilized spike glycoprotein ectodomain (S2P, red) fusing with the NDV fusion protein transmembrane and cytoplasmic tail domain (Ftmct, blue). The bottom linear diagram shows the coexpression construct, the full-length NDV matrix protein (NDV-M, blue) and nucleoprotein (NDV-NP, blue), linked by a self-cleaving peptide (P2A). (**B**) Generation of the HEK293T cell pool that stably expressed S2P on the cell surface. HEK293T cells were cotransfected with pS2P-NDV-Ftmct and pCMV6-A-puro plasmids. S2P-expressing cells were live immunostained with the anti-SARS-CoV-2 spike-specific neutralizing antibody S652-118 and AF488-conjugated secondary antibody then live sorted using FACS. (**C**) HEK293T cell pool that stably expressed the S2P glycoprotein on the cell surface was confirmed by live immunostaining with antibody S652-118 (left) and without (right). (**D**) Immunoblot analysis of S2P-NDVLP with S2P glycoprotein-immunized mouse sera at Week 5 compared with the S2P glycoprotein. See Figure S2 for details on quantification. (**E**) Structural and morphological characterization of VLPs by transmission electron microscopy. Negative staining showing well-defined S2P-NDVLP (80 nm) with long-length spikes (~17 nm) and short-length spikes protruding at variable angles from the S2P-NDVLP surface (red color arrows in the larger box, top left corner).

The DNA sequence encoding the S2P-NDV-Ftmct chimeric protein fusing the ectodomain (residues 1–1208) of SARS-CoV-2, pre-fusion-stabilized spike by two proline substitutions at residues 986–987 (S2P), and furin cleavage site abolished by GSAS substitution at residues 682–685 (GenBank: MN908947) [41,42], with the NDV fusion protein (F, Avian avulavirus 1, GenBank: AMQ09764) transmembrane and cytoplasmic domains (Ftmct, residues 499–553) (Figure 1A), was synthesized by GeneImmune Biotechnology and inserted into pVRC8400 to construct plasmid pS2P-NVD-Ftmct. For the construction of pNDV-P2A-NP, the DNA sequence encoding NDV matrix protein residues 1–364 (M, Avian avulavirus 1, GenBank: AMQ09763) and nucleoprotein residues 2–489 (NP, Avian avulavirus 1, GenBank: AMQ09761) in a single transcript, with the two proteins separated by P2A, a self-cleavage peptide sequence [43] during post-translation processing, was synthesized and inserted into vector pVRC8400.

### 2.3. Preparation of HEK293T Cell Pool Stably Expressing the SARS-CoV-2 Spike (S2P)

Log-phase (5 × 10^6^) growing HEK293T cells were seeded in a T75 cm^2^ flask (High Binding Surface, Corning, NY, USA) and cultured at 37 °C and 5% CO_2_ for 24 h at about 70% cell confluency. Prior to transfection, the culture medium was replaced with 7 mL of fresh optimized expression medium (RealFect-Medium, ABI Scientific). A 24 μg amount of pS2P-NDV-Ftmct and 6 μg of pCMV6-A-puro (a vector containing a puromycin resistance gene purchased from ORIGEN) was incubated with 90 μL of TrueFect-Max transfection reagent (United BioSystems, Bethesda, MD, USA) in 1.5 mL of serum-free Opti-MEM (Thermo Fisher Scientific) for 15 min at room temperature (RT) then added to cells in T75 cm^2^ flask. Transfection was incubated at 37 °C and 5% CO_2_ for 12 h, then transfected cells were fed with 6 mL of enriched expression medium (CelBooster Cell Growth Enhancer Medium for Adherent cell, ABI Scientific) containing 3× Streptomycin–Penicillin. Twenty-four hours post-transfection, transfected cells were selected by 10 μg/mL puromycin in culture medium and cultured for 10 days, with cell passages every 3 days. The adherent selected cells were dissociated with 0.05% trypsin–EDTA (Thermo Fisher Scientific) and incubated in suspension medium (40% DMEM, 30% CelBooster, 30% 293 Freestyle Medium (Thermo Fisher Scientific)) in a shake flask at 37 °C, 8% CO_2_, and 120 rpm overnight. The suspended cells were collected by centrifugation at 200× *g* and stained with SARS-CoV-2 spike-specific antibody S652-118 [41] at 10 μg/mL in RealFect-Medium and at 4 °C in a fridge for 30 min, followed by staining with 1:250 diluted AF488-conjugated secondary antihuman IgG (Thermo Fisher Scientific) in the dark and at 4 °C in a fridge for 20 min. Following washes, the positive cells expressing a spike on the cell surface were sorted using the FACS Cell Sorter (FACSAria II, BD, Franklin Lakes, NJ, USA) and immediately cultured in a T75 cm^2^ flask in DMEM medium containing 20% FBS and 2× Streptomycin–Penicillin. The selected cells were incubated without primary antibody S652-118, with AF488-conjugated secondary antihuman IgG alone as a negative control. The FACS-sorted cells expressing a spike on the cell surface were expanded as a cell pool and used for VLP production.

### 2.4. Live Cell-Surface Staining

Human-fibronectin-coated 6-well cell culture plates (High Binding Surface, Corning) were prepared via incubation with a human fibronectin working solution at 2 μg/mL (Corning) in a fridge overnight, washed, and dried in a cell culture hood. Log-phase (5 × 10^5^/well) growing HEK293T cells stably expressing the SARS-CoV-2 spike were seeded in 6-well cell culture plates precoated with human fibronectin and cultured at 37 °C and 5% CO_2_ for 26 h at about 70% cell confluency. Live cells were stained with antibody S652-118 at 10 μg/mL in RealFect-Medium and incubated at 37 °C and 5% CO_2_ for 30 min. After gently washing twice with warmed RealFect-Medium, live cells were further incubated with 1:250 diluted AF488-conjugated secondary antihuman IgG in the dark at RT for 20 min, followed by gentle washing three times with RealFect-Medium or 1× PBS plus 1% BSA. The SARS-CoV-2 spike expressed on the cell surface was imaged under a fluorescent microscope.

### 2.5. Production and Purification of SARS-CoV-2 S2P-NDVLP

Log-phase (11 × 10^6^) growing HEK293T cells stably expressing the spike were seeded in a T150 cm^2^ flask and cultured at 37 °C and 5% CO_2_ for 24 h at 70–80% cell confluency. Prior to transfection, the culture medium was replaced with 15.5 mL of fresh RealFect-Medium. A 24 μg amount of pNDV-M-P2A-NP DNA and 48 μL of P3000 were incubated with 72 μL of Lipofectamine 3000 transfection reagent (Thermo Fisher Scientific) in 2.3 mL of serum-free Opti-MEM for 15 min at RT then added to cells in a T150 cm^2^ flask, according to the manufacturer’s instructions. Transfected cells were incubated at 37 °C and 5% CO_2_ for 12 h and then fed with 7 mL of CelBooster Cell Growth Enhancer Medium containing 3× Streptomycin–Penicillin. The culture supernatant from transfected cells was harvested three times on Days 1.5, 3.0, and 5.0 post-transfection. SARS-CoV-2 S2P-NDVLPs were purified by pelleting and sucrose gradient ultracentrifugation as follows: the harvested supernatant was clarified by centrifugation at 1500× *g* at 4 °C for 30 min, followed by filtration through a 0.8 μm filter. The supernatant was transferred into a 36 mL ultracentrifuge tube (Beckman, CA), with 3 mL of 20% sucrose in MES buffer (150 mL NaCl, 20 mM MES, pH 6.0) on the bottom to pellet the raw VLP particles by centrifugation at 20,000 rpm at 4 °C for 3 h (Sorvall SureSpine 630 rotor, Thermo Fisher). The particle pellet was responded in 3 mL of MEB buffer and laid on top of the discontinuous sucrose gradient in a 12 mL ultracentrifuge tube (Beckman), with 65% sucrose on the bottom and 20% sucrose on the top and a total of 6 layers (65%, 50%, 40%, 35%, 30%, and 20%, 1.5 mL/layer), and ultracentrifuged at 36,000 rpm at 4 °C for 6 h (Beckman SW28.1 rotor). S2P-NDVLP was sedimented in a 50% sucrose layer and determined by immune dot blotting with patient convalescent plasms or S2P-immunized mouse sear as well as negative-staining electron microscopy. The concentration of S2P-NDVLP was measured with the Micro BCA Protein Assay Kit (Thermo Fisher Scientific). The amount of S2P of S2P-NDVLP was determined by quantification of S2P immunoblot signals.

### 2.6. Negative-Staining Electron Microscopy

The sample was applied without dilution to a freshly glow-discharged carbon-coated copper grid. After a 15 s incubation period, the extra sample was removed using blotting paper, and the grid was washed consecutively with three drops of buffer containing 10 mM HEPES, pH 7.4, and 150 mM NaCl. Objects that adsorbed the carbon were negatively stained with 0.75% uranyl acetate, and the grid was allowed to air dry. Images were taken using a Hitachi H-7650 transmission electron microscope operated at 80 kV.

### 2.7. Preparation of Prefusion-Stabilized SARS-CoV-2 Spike Glycoprotein (S2P Trimer) and RBD Glycoprotein

The expression plasmid designs and protein production of the S2P trimer glycoprotein and RBD were performed as described previously [41]. Briefly, each protein sequence was fused with an 8-mer His-tagger sequence at the carboxyl terminus, and the DNA encoding code was optimized and synthesized by GeneImmune Biotechnology. Each His-tagged protein was expressed in 293 Freestyle cells by transient transfection for 6 days at 37 °C. On Days 1 and 3 post-transfection, enriched feed medium, Cell Growth Enhancer for suspension cells (ABI Scientific), was added into the culture at a 5% culture volume. Following expression, the cell supernatant was collected, filtered, and applied to a nickel affinity resin. The protein-bound resin was washed extensively with an increasing imidazole concentration and eluted with 300 mM imidazole buffer. His-tagged HRV3c protease was added to the nickel elution and incubated overnight at 4 °C. The cleaved glycoprotein was then concentrated using spin filters and applied to a Superdex S-200 gel filtration column, equilibrated in PBS. The S2P glycoprotein eluted primarily in a single peak corresponding to a trimeric size, with RBD as a monomer. The single-peak fractions were collected, concentrated to 1 mg/mL, and flash-frozen in liquid nitrogen prior to storage at −80 °C.

### 2.8. Preparation of Antibodies

All antibodies used in the study were produced as described previously [41].

### 2.9. Antigenic Analysis

The antigenicity of S2P-NDVLP compared to the S2P soluble protein was assessed by biolayer interferometry (BLI) assay and ELISA. For the BLI assay, experiments were performed with a FortéBio Octet HTX instrument (FortéBio) in a tilted black 384-well plate (Geiger Bio-One) at 30 °C, with constant plate agitation at 1000 rpm. Samples were diluted in PBS + 1% BSA. The antibody at 40 μg/mL was immobilized on antihuman IgG Fc capture biosensors (FortéBio) for 300 s. Binding to various concentrations of S2P or S2P-NDVLP was measured for 300 s. Two-fold serial dilutions yielded a concentration series for S2P (234.08–3.66 nM) and S2P-NDVLP (9.36–0.15 nM). Dissociation was recorded for 300 s. Data Analysis Software v12.0 (Fortebio) was used to analyze the raw data. Double-reference subtraction was performed for the S2P-NDVLP samples to correct for nonspecific binding and buffer mismatch. Nonspecific binding was negligible for the S2P sample, and reference well subtraction was performed to correct for systematic baseline drift. Kinetic analysis was applied to the data using a global fitting and 1:1 binding model.

For the ELISA, 96-well ELISA plates (Nunc Maxisorp, Thermo Fisher Scientific) were coated with 10 μg/well of Lectin (Galanthus Nivalis, SIGMA) in PBS overnight at 4 °C and blocked with a standard block (1% BSA and 0.05% Tween in PBS). Serially diluted S2P-NDVLP or S2P soluble glycoprotein were incubated in precoated Lectin-plates for 1.5 h at RT. Following washes, antibodies at 10 μg/mL were incubated in the plates for 50 min at RT. Bound antibodies were then detected by a goat antihuman IgG Fc fragment–horseradish peroxidase (HRP) conjugate (Jackson Immunoresearch), according to the manufacturer’s instructions, for 30 min at RT, followed by the HRP substrate, *3,5,3′5′-tetramethylbenzidine (TMB)* (BioFX TMB, SurModics), for 10 min at RT. The reaction was stopped with 0.5 N sulfuric acid. The optical density (OD_450_) was read at 450 nm with a spectrophotometer (SpectroPlus, Molecular Devices). EC_50_ titers were determined using a log (agonist) vs. normalized response (variable slope) nonlinear function in the GraphPad Prism v8 software (GraphPad Software, CA, USA).

### 2.10. Quantitation of SARS-CoV-2 Spikes of SARS-CoV-2 S2P-NDVLP

The amount of S2P spikes of S2P-NDVLP compared to the S2P soluble glycoprotein alone was determined by quantification of the Western Blot signal density. Briefly, all samples were mixed with an equal volume of SDS sample buffer (reducing) and heated at 95 °C for 5 min. NuPage SDS-PAGE gels (10 wells) were used to separate proteins. Gels were transferred to a PVDF membrane using the Trans-Blot Turbo Transfer System (BioRad) for 7 min at 25 V. The membrane was blocked with 10 mL of PBS-T (0.05% Tween 20) and 5% (*w*/*v*) powdered milk (Thermo Fisher Scientific) for 1 h at RT using an orbital shaker and incubated with serum from mice immunized with S2P at Week 5, diluted 100-fold in PBS-T for 1 h and further with a secondary HRP-conjugated goat antimouse IgG+IgM antibody (Jackson ImmunoResearch Laboratories) at a dilution of 1:2000 for 1 h. Signals were developed with West Pico solution (Thermo Fisher Scientific) for 10 min at room temperature and imaged on a Chemidoc (BioRad) using the default settings for chemiluminescence. Quantification of band intensity was performed using the ImageJ software (NIH). Intensities from the S2P soluble glycoprotein dilutions were plotted against the known concentrations, and linear regression was used to fit the data. The slope of the standard curve was used to estimate the amount of S2P in the S2P-NDVLP samples. The S2P amount presented on S2P-NDVLP was calculated as 0.25% of the VLP.

### 2.11. Mouse Immunization and Ethics Statement

Animal procedures, housing, and care were performed in accordance with local, state, and federal policies. Mouse experiments were carried out in compliance with regulations and guidelines from the Animal Care and Use Committee of the Vaccine Research Center, National Institute of Allergy and Infectious Diseases, National Institutes of Health (ASP code, VRC-19-0799). Six-week-old female BALB/cJ mice (Jackson Laboratories) were immunized intramuscularly at Weeks 0 and 3 with either the soluble tag-free SARS-CoV-2 S2P glycoprotein (0.4, 2, and 10 μg) or S2P-NDVLP (2, 10, 50, and 250 μg) adjuvanted with Sigma Adjuvant System (SAS) (Sigma-Aldrich), as previously described [21,44]. Mice were bled at Weeks 0, 2, and 5 for preimmunization, postprime, and postboost serological analyses, respectively.

### 2.12. Serum IgG Assay

Nunc Maxisorp 96-well ELISA plates (Thermo Fisher Scientific) were coated with 100 ng/well of the antigen overnight at 4 °C. Standard blocks and washes were applied. Heat-inactivated murine sera were serially diluted and incubated in precoated plates for 1 h at RT. Following washes, antibody binding was detected using antimouse IgG, IgG1, or IgG2a-horseradish peroxidase conjugates (Thermo Fisher Scientific), according to the manufacturer’s instruction. Plates were then developed with 3,5,3′5′-tetramethylbenzidine (TMB) (KPL) substrate, and the reaction was stopped with 1 N sulfuric acid. The resulting signals read at OD_450/650_ were used to calculate endpoint titers, defined as the dilution that emitted an optical density exceeding a 4× background (secondary antibody alone). EC_50_ titers were determined as the serum titers using a log (agonist) vs. normalized response (variable slope) nonlinear function in GraphPad Prism v8.

### 2.13. Pseudovirus Neutralization Assay

The SARS-CoV-2 pseudovirus was generated using a lentiviral-based system. A codon-optimized plasmid CMV/R-SARS-CoV-2-encoding D614G spike variant was constructed and cotransfected with a lentivirus backbone, luciferase reporter, and human transmembrane protease serine 2 (TMPRSS2) in HEK293T/17 cells (ATCC #CRL-11268). The pseudovirus was harvested 72 h post-transfection, clarified, and stored at −80 °C before use. The SARS-CoV-2 pseudovirus was mixed with heat-inactivated murine sera and incubated for 45 min at 37 °C. Sera dilutions were then applied to stably transfected 293T cells overexpressing ACE2, and the luciferase signal was surveyed after 72 h. Percent neutralization was defined as the luciferase activity of uninfected cells as 100% neutralization and cells infected with only the pseudovirus as 0% neutralization. ID_50_ titers were determined as the serum titers that gave 50% neutralization using a log (agonist) vs. normalized response (variable slope) nonlinear function in GraphPad Prism v8.

### 2.14. Serum Anti-RBD or Anti-S2P Titer Assay

Mouse sera were assessed for binding to S2P or RBD by BLI assay with a FortéBio Octet HTX instrument. Sera were diluted 1:100 in 1% BSA/PBS. Streptavidin (SA) biosensor tips (FortéBio) were equilibrated in 1% BSA PBS before the assays and then loaded with the biotinylated S2P or RBD at 40 μg/mL in 1% BSA/PBS for 300 s, followed by equilibration in buffer for 60 s. The sera responses were measured by the association step for 300 s in sera, followed by a dissociation step for an additional 120 s. The prebleed sera response for each group of immunogens was used as a reference. BLI RBD-binding responses were normalized and analyzed with the GraphPad Prism v8 software.

### 2.15. Statistical Analyses

All experimental result data were subjected to statistical analyses using the GraphPad Prism v8 software and a Student’s *t*-test. Multiple comparisons between groups were calculated using one-way ANOVA in GraphPad Prism v8. The Kruskal–Wallis test was used to test differences between the groups, and geometric means were indicated with a red bar. Spearman correlation was used to test the correlations between neutralization and antibody binding. EC_50_ or ID_50_ values were calculated using nonlinear, dose–response regression analysis.

## 3. Results

### 3.1. Design of S2P-NDVLP Constructs and Generation of S2P-NDVLP

The SARS-CoV-2 spike glycoprotein is a key antigen to mediate protective immune responses [45,46]. To engineer a nonreplicating membrane-encapsulated VLP displaying SARS-CoV-2 spikes on its surface, we exploited the NDVLP technology [38,39]. We prepared a chimeric construct, S2P-NDV-Ftmct, containing SARS-CoV-2 prefusion-stabilized spike ectodomain (S2P, residues 1–1208) [42] fused with the transmembrane and cytoplasm tail domains of NDV fusion protein (NDV-Ftmct, residues 499–553) (Figure 1A, top). Another construct, NDV-M-P2A-NP, was prepared by linking NDV matrix protein (NDV-M, residues 1–364) with nucleoprotein (NDV-NP, residues 2–489) through a self-cleavage peptide sequence (P2A) (Figure 1A, bottom). The plasmid pS2P-NDV-Ftmct was cotransfected with a drug-selection plasmid pCMV6-A-Puro into HEK293T cells. After incubating in a puromycin selection medium for ten days, the cells stably expressing S2P on the cell surfaces were separated by the FACS living cell surface sorting method (Figure 1B) and further cultured as a stable cell pool. Live-cell immunofluorescence staining with a SARS-CoV-2 spike-specific antibody S652-118 confirmed that S2P was expressed on the cell surfaces (Figure 1C), as these untreated live 293T cells are nonpermeable to the IgG antibody. Further transfection of the pNDV-M-P2A-NP plasmid into S2P-expressing stable HEK293T cells resulted in a high titer of S2P-NDVLP secreted into the cell culture supernatant. S2P-NDVLP was pelleted, followed by a continuous sucrose gradient (20% to 65% sucrose) through ultracentrifugation. S2P-NDVLP was localized in the ~50% sucrose fraction, detected by immunoblotting assay with anti-SARS-CoV-2 sera obtained from COVID-19 patients or by VLP capture ELISA with the neutralizing antibody S652-118 (Figure S1A,B). In addition, purified S2P-NDVLP specifically reacted with S2P glycoprotein-immunized mouse sera (Figure 1D). Negative-stain electron microscopy (EM) revealed that purified S2P-NDVLPs were approximately spherical, with diameters of 50 nm to 90 nm to the outside of the lipid bilayer of sizes and decorated with long (17.4 ± 2.3 nm) and short spikes (Figure 1E), structurally similar to the spikes on the surface of SARS-CoV-2 virions [47]. The EM images also showed broken S2P-VLPs with aggregated proteins, presumably due to VLP pelleting. To calculate immunization dosage, we measured the total S2P-NDVLP glycoprotein concentration by BCA assay and quantified the S2P amount in the purified S2P-NDVLP sample by Western blot to be about 0.25% of the total S2P-NDVLP glycoprotein by weight (Figure S2).

### 3.2. S2P-NDVLP Was Specifically Recognized by SARS-CoV-2-Neutralizing Antibodies

**Figure 2:**
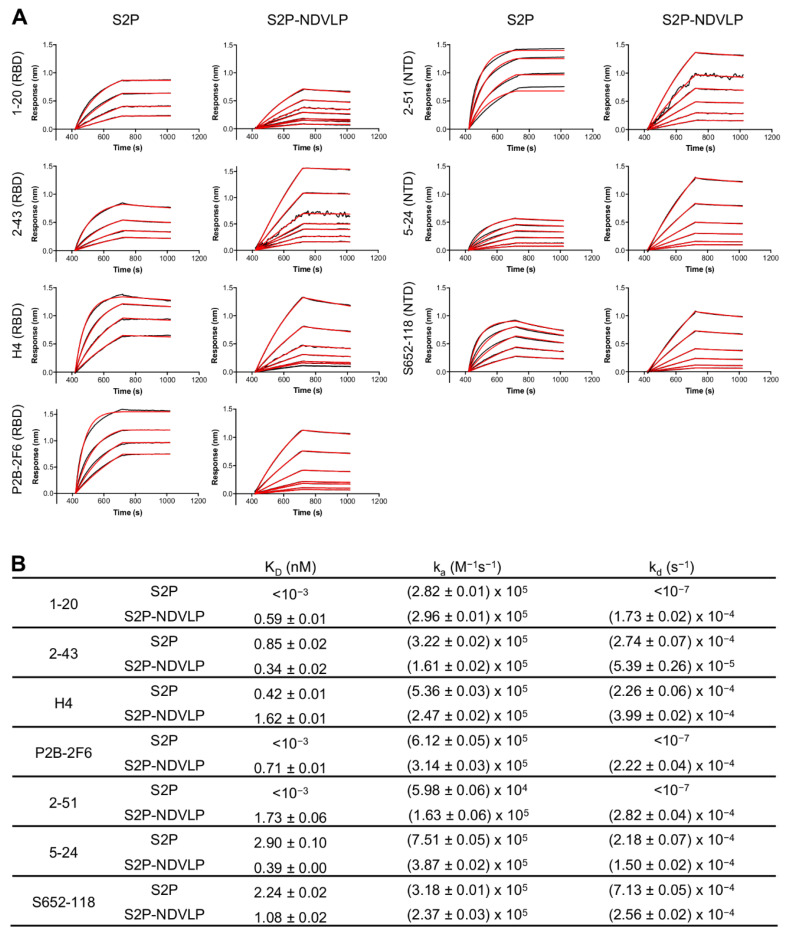
Characterization of S2P-NDVLP antigenicity with a panel of SARS-CoV-2 spike-specific neutralizing antibodies. (**A**) Biolayer interferometry (BLI)-binding curves. Receptor-binding domain (RBD)-specific neutralizing antibodies: 1-20, 2-43, H4, and P2B-2F6; NTD-specific neutralizing antibodies: 2-51, 5-24, and S652-118. Binding kinetics were determined by biolayer interferometry. Kinetics curves were obtained using an Octet HTX instrument with a series of concentrations (9.36–0.15 nM for S2P-NDVLP and 234.08–3.66 nM for S2P). (**B**) Kinetic parameters determined from BLI-binding curves. Data Analysis Software v12.0 (Fortebio) was used to analyze the raw data and the calculated K_D_, k_a_, and k_d_ values. Kinetic analysis was applied to the data using a global fitting and a 1:1 binding model.

To assess the antigenic specificity of S2P-NDVLP, we analyzed its binding to a panel of SARS-CoV-2-neutralizing antibodies, including NTD-specific 2-51, 5-24, and S652-118 and RBD-specific H4, P2B-2F6, 1-20, and 2-43 [41,48,49,50], by biolayer interferometry (BLI) (Figure 2). The binding kinetics of S2P-NDVLP with the antibodies were based on the molar concentration of S2P in S2P-NDVLP, as measured by Western blotting quantification (Figure S2). The results showed that S2P-NDVLP bound to all test SARS-CoV-2-neutralizing antibodies with kinetic parameters that were generally similar to those of soluble S2P, except for NTD-binding antibody 2-51 and RBD-binding antibodies P2B-26 and 1-20, in which the binding off-rates (k_d_) were lower than the BLI detection limits. Overall, the binding on-rates (k_a_) were similar to the corresponding values for S2P, with less than a three-fold difference; the measurable off-rates were also similar, within a four-fold difference. These results suggested that S2P spikes presented on S2P-NDVLP were structurally and conformationally similar to the soluble S2P glycoprotein. Importantly, a substantial avidity effect versus soluble S2P was not observed, suggesting spikes to be somewhat sparsely displayed on the surface of VLP, consistent with the lower amount of S2P present on S2P-VLP, as quantified by Western blot.

### 3.3. Immunogenicity Assessments of S2P-NDVLP in Mice

To evaluate the immunogenicity of S2P-NDVLP in comparison to the soluble S2P glycoprotein, we immunized BALB/cJ mice with S2P-NDVLP (2 µg, 10 µg, 50 µg, or 250 µg) or the S2P soluble glycoprotein (0.4 µg, 2 µg, or 10 µg) at Week 0 (prime) and Week 3 (boost). Immunogens were adjuvanted with Sigma Adjuvant System (SAS), a TLR4 agonist. The S2P-NDVLP doses were equivalent to 0.005, 0.025, 0.125, and 0.625 µg of S2P, as determined by S quantification (Figure S2). A 250 µg amount of S2P-NDVLP (Group 4) contained 0.625 µg of S2P, which was the closest to the lowest S2P glycoprotein dose (Group 5).

**Figure 3:**
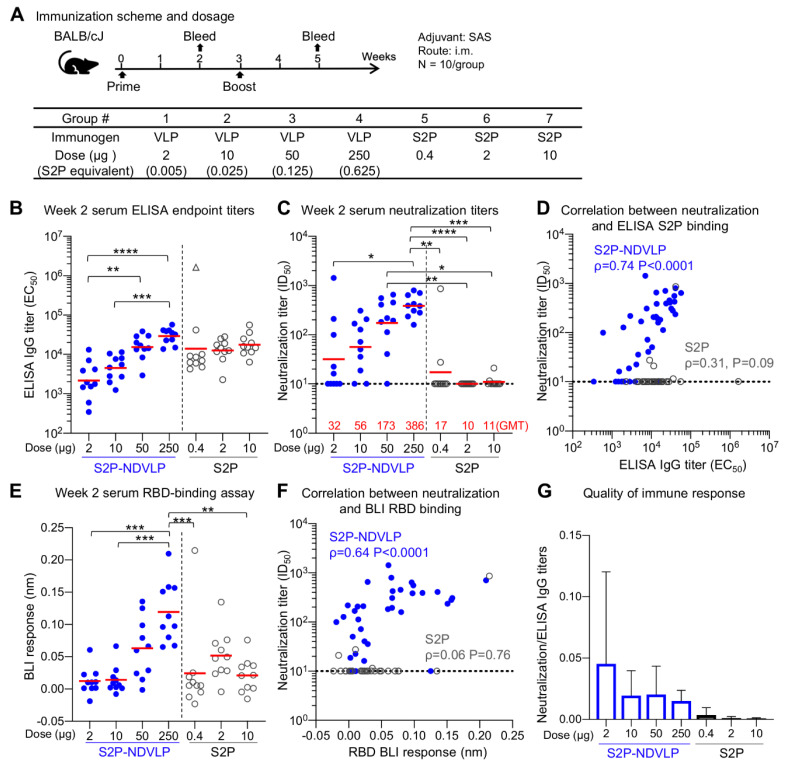
Single immunization of S2P-NDVLP elicited a high immune response against SARS-CoV-2 virus in mice at Week 2. (**A**) Scheme of the immunization regimen and groups. The control immunogen was a trimeric spike S2P glycoprotein. The amount of S2P in the S2P-NDVLP immunogen was quantified by Western blotting with S2P-immunized mouse sera (see Figure S2). (**B**) S2P-NDVLP elicited substantial IgG titers against the SARS-CoV-2 spike at Week 2. The endpoint IgG titers were measured, and EC_50_ values were calculated as the readout for ELISAs with Prism. The triangle symbol indicates the ELISA titer at assay maximum. (**C**) S2P-NDVLP elicited substantial SARS-CoV-2 pseudovirus neutralization titers at Week 2. Serum-neutralizing antibodies against a D614G pseudovirus were measured, and ID_50_ values were calculated as the readout of the neutralization assay with Prism. The geometric mean titer (GMT) of each group is marked in red numbers on the horizontal axis. (**D**) Correlation between neutralization titers and ELISA S2P-specific IgG titers existed in the S2P-NDVLP-immunized groups but not in the S2P-immunized groups. (**E**) RBD-specific serum antibody titers were measured as BLI responses and calculated with Prism. The titers among the S2P-NDVLP-immunized groups were dose dependent at Week 2. (**F**) Correlation between neutralization titers and BLI RBD-specific antibody titers existed in the S2P-NDVLP-immunized groups but not in the S2P-immunized groups. (**G**) Quality of immune responses elicited by immunization was calculated as the ratio of neutralization titer to total ELISA IgG titer, plotted as mean and SD. The horizontal dotted line in (**C**–**F**) represents the low detection limit for the neutralization titer. In (**B**–**E**), the Kruskal–Wallis test was used to test differences between the groups, and P-values were designated as *: *p* < 0.05; **: *p* < 0.01; ***: *p* < 0.001; ****: *p* < 0.0001. Geometric means were indicated with a red bar. Spearman correlation was used in (**D**,**F**).

**Figure 4:**
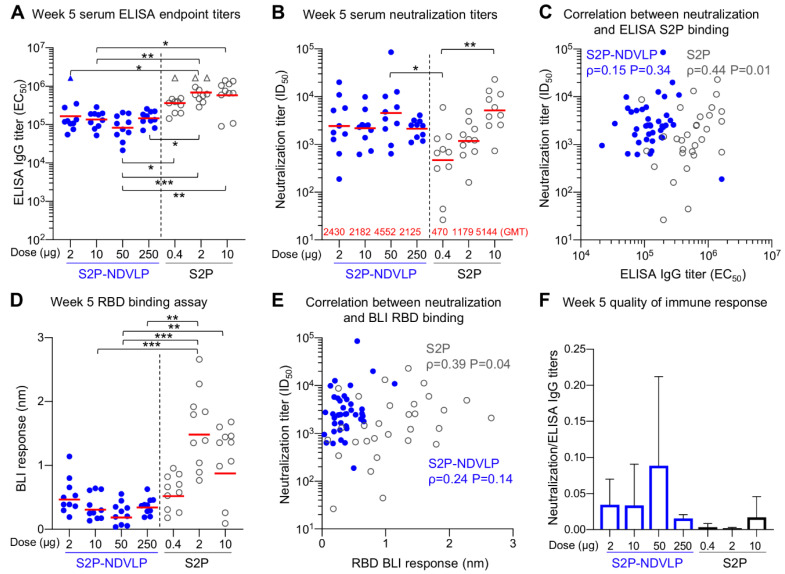
S2P-NDVLP elicited a robust anti-SARS-CoV-2-neutralizing response with a relatively low S2P-equivalent dose in mice at Week 5. (**A**) Boost immunization elicited higher ELISA titers against the SARS-CoV-2 spike at Week 5. The titers among the S2P-NDVLP-immunized groups were not significantly different. IgG titers in the S2P-immunized groups were significantly higher than the S2P-NDVLP-immunized groups. Triangle symbols indicate the ELISA titers at assay maximum. (**B**) The S2P-NDVLP groups had robust neutralization activities at Week 5 but were not significantly different from each other. Serum neutralization titers (ID_50_) were measured against a SARS-CoV-2 D614G variant pseudovirus. (**C**) Correlation between neutralization titers and ELISA S2P-specific IgG titers did not exist in the S2P-NDVLP-immunized groups, but a weak correlation existed in the S2P-immunized groups. (**D**) RBD-specific serum antibody titers were measured as BLI responses and calculated with Prism. BLI RBD-specific serum antibody titers in the 2 and 10 μg S2P-immunized groups were significantly higher than all other groups. (**E**) Correlation between neutralization titer and BLI RBD-specific antibody titer did not exist in the S2P-NDVLP-immunized groups, but a weak correlation existed in the S2P-immunized groups. (**F**) Quality of the immune responses elicited by immunization was calculated as the ratio of the neutralization titer to the total ELISA IgG titer, plotted as the mean and SD. In (**A**,**B**,**D**), the Kruskal–Wallis test was used to test differences between the groups, and P-values were designated as *: *p* < 0.05; **: *p* < 0.01; ***: *p* < 0.001; ****: *p* < 0.0001, and geometric mean titers were marked with a red bar. Spearman correlation was used in (**C**,**E**).

Mouse sera were analyzed for SARS-CoV-2 S-specific IgG titers by ELISA (Figure 3B and Figure 4A). Postprime, both S2P-NDVLP and S2P elicited sufficiently high spike-specific IgG titers. The titers among the S2P-NDVLP-immunized groups were dose dependent, whereas, for the S2P glycoprotein groups, dose dependency was not detected. S-specific IgG titers in 250 µg S2P-NDVLP-immunized mice (geometric mean titer (GMT) = 35,000) trended two-fold higher than that in 10 µg S2P-immunized mice (GMT = 17,484), indicating single immunization of S2P-NDVLP could be sufficient to elicit a high humoral immune response. Boosting in the S2P-NDVLP-immunized mice increased spike-specific IgG titers by six-fold (Week 5 GMT = 210,000 vs. Week 2 GMT = 35,000). In contrast, boosting with the S2P glycoprotein resulted in a nearly 50-fold increase in spike-specific IgG, rendering postboost S-specific IgG levels significantly higher in the S2P-immunized mice as compared to the S2P-NDVLP-immunized groups. Neither immunogen’s ability to elicit S-specific IgG following boost was dose dependent (Figure 4A).

Historically, vaccine-associated enhanced respiratory disease (VAERD), which is associated with T helper 2 cell (T_H_2)-biased immune responses, has been shown in some animal models following immunization with whole-inactivated vaccines and other types of experimental SARS-CoV vaccines [51,52,53]. Previously, we showed that SAS-adjuvanted S2P elicited balanced T_H_1–T_H_2 responses [21]. Similarly here, following two doses of S2P-NDVLP or S2P, we compared levels of S-specific IgG2a and IgG1, which are the respective surrogates of T_H_1 and T_H_2 responses. As expected, due to adjuvant choice, both immunogens elicited S-binding antibodies in the IgG2a and IgG1 subclasses, indicating a balanced T_H_1–T_H_2 response and avoidance of VAERD-linked T_H_2-bias. (Supplementary Figure S3).

Next, to gauge antibody neutralization capacity, serum neutralization activity against the predominantly circulating SARS-CoV-2 D614G pseudovirus was assessed (Figure 3C and Figure 4B). Notably, detectable neutralizing titers were elicited by single immunization of S2P-NDVLP (Groups 2, 3, and 4 with a GMT of 56, 173, and 386, respectively), in a dose-dependent manner (Figure 3C). S2P immunization generally did not elicit detectable neutralizing antibodies following one dose. Both 50 and 250 µg of S2P-NDVLP elicited significantly higher ID_50_ titers than all the S2P-immunized groups (Figure 3C). There was a weak correlation between neutralization titers and S2P-specific IgG titers in S2P-NDVLP-immunized groups, whereas, in the S2P-immunized groups, there was no correlation despite substantial ELISA titers at Week 2 (Figure 3D). To determine antibody responses related to serum neutralization, we assessed serum binding to RBD by BLI (Figure 3E). We observed in general lower BLI responses for the S2P-immunized groups at Week 2. Although there was no correlation for the S2P groups between RBD-binding responses and neutralization titers due to lack of neutralization of most sera, the single mouse in the 0.4 µg S2P-immunized group with substantial neutralization titer also had the highest RBD BLI response (Figure 3F). The neutralization titers of the S2P-NDVLP-immunized groups exhibited a stronger correlation with RBD-binding responses than with the antispike ELISA titers. This correlation between serum RBD-specific antibody responses and neutralization titers was consistent with a recent study showing that RBD-specific antibody titers correlate with neutralizing antibody titers in convalescent sera [54]. We analyzed the quality of the immune responses by calculating the ratio of neutralization titers to the antispike ELISA titers (Figure 3G); the S2P-NDVLP-immunized groups had higher neutralization to ELISA titer ratios than the S2P-immunized groups. Together, these data suggest that the VLP-based immunogen elicits a functionally better antibody response.

Postboost, we observed improved neutralizing responses relative to Week 2 in all groups, especially for the S2P-immunized groups (Figure 4B). The overall neutralizing titers increased about 20-fold in the S2P-NDVLP-immunized mice (GMT = 4552), and all four S2P-NDVLP-immunized groups had similar GMT neutralization titers, including the 2 µg S2P-NDVLP-immunized group, for which the dose was equivalent to 0.005 µg of the S2P glycoprotein. In contrast, a more than 100-fold increase in neutralization titer was observed in the S2P-immunized mice. Further, dose dependency was exhibited; 10 µg of the S2P glycoprotein elicited significantly more neutralizing antibodies than 0.4 µg. In addition, boosting revealed similar neutralization titers in the S2P-NDVLP- and S2P-immunized mice. In contrast to postprime, there was no correlation between neutralization titer and S2P-specific IgG titer following boost in the S2P-NDVLP-immunized mice; oppositely, a weak correlation existed in the S2P-immunized mice (Figure 4C). RBD-specific serum antibody titers in the 2 and 10 µg S2P-immunized mice were significantly higher than all other groups (Figure 4D). Similar to our finding with S-specific antibodies, there was no correlation between neutralization titers and RBD-specific antibody titers in the S2P-NDVLP-immunized mice and a weak correlation in the S2P-immunized mice (Figure 4E). Furthermore, the S2P-NDVLP groups still exhibited a higher ratio of neutralization titer to ELISA titer than most of the S2P groups at Week 5 (Figure 4F), again indicating a functionally better antibody response.

Overall, the S2P-NDVLP-immunized mice showed dose-dependent spike-specific IgG titers or pseudovirus neutralization titers at Week 2 among all dosage groups (ranges: 2 μg–250 μg), suggesting that the S2P-NDVLP dosages were not saturating during the prime immunization; however, by Week 5, both IgG and neutralization titers were similar, suggesting even the 2 ug dose to be saturating. By contract, all groups of S2P-immunized mice showed a similar level of spike-specific IgG titers at Weeks 2 or 5 without dose dependency (ranges: 0.4 μg–10 μg), with dose dependency only observed for neutralization titers at Week 5. These results suggest different factors to impact IgG and neutralizing titers at Weeks 2 and 5 and are consistent with quantification indicating the level of S2P in the S2P-NDVLP to be ~400-fold lower than S2P glycoprotein at the same overall protein concentration. Thus, S2P-NDVLP can be effective at eliciting substantial neutralizing titers two weeks after a single immunization, and a much lower S2P-equivalent dose of S2P-NDVLP can elicit similar neutralizing responses at a high dose of the soluble S2P trimer at Week 5 by boost immunization.

## 4. Discussion

In this study, we applied NDVLP technology to develop an immunogen that displayed prefusion-stabilized membrane-anchored SARS-CoV-2 spikes on the surface of a Newcastle disease virus-like particle. NDVLP-displayed spikes were in a prefusion conformation, were generally similar antigenically to soluble S2P trimers, and elicited substantial neutralizing titers in mice. These titers appeared to be improved over a previously described NDVLP displaying a nonstabilized version of the spike [9], although the comparison is complicated by differences in immunization regimens. We also observed improved immunogenicity of S2P-NDVLP over a soluble S2P trimer after a single immunization, an improvement related to S2P-NDVLP characteristics. The increased size of the VLP versus the trimer should enhance its uptake by dendritic cells; the multivalent presentation of spike antigens on a single particle should enable a single S2P-NDVLP particle to bind and activate multiple B-cell receptors; the S2P-NDVLP-lipid membrane may allow for increased mimicry of the spikes on authentic SARS-CoV-2 spike; and many more T-cell epitopes are present in the S2P-NDVLP particle versus the S2P trimer, enabling T help after a single priming immunization. We note that the boost immunization of S2P-NDVLP increased anti-SARS-CoV-2 pseudovirus-neutralizing titers, but the differences among the mice groups were not significant, suggesting a potential plateau or neutralization titer limit for S2P-NDVLP immunization.

In agreement with the findings obtained with a prime immunization of S2P-NDVLP, sufficient SARS-CoV-2-neutralizing activity elicited after two or four weeks has been observed for some S2P spike-presented viral-vector-based or virus-derived replicon RNA-based vaccine candidates in mice [8,29,30,31]. The general similarity in antigenicity between S2P-NDVLP and soluble S2P (Figure 2) suggests the S2P spike presented on S2P-NDVLP to be similar to that of the soluble S2P trimer. However, attachment of the spike on the membrane surface of VLP does restrain its mobility and alter its surface accessibility; specifically, the apex of the spike, where RBD and NTD epitopes are located, is likely to be more accessible than the base when attached to VLP. The multimeric display is also likely to impact recognition by bivalent immunoglobulin, and we indeed observed lower off-rates for some neutralizing antibodies. Overall, VLP vaccines have emerged as potent inducers of antibody and helper T-cell responses [55,56], and these characteristics likely explain the improved immunogenicity of S2P-NDVLP.

Consistent with the observation from the control S2P-immunized mice in our study, immunization studies with SARS-CoV-2 S2P vaccine candidates have shown that single immunization is often unable to elicit high titer neutralizing activity in immunized mice at Week 2, even though reasonable titers of total spike-specific IgG have been observed (EC_50_ > 1 × 10^4^) [21]. The mRNA-1273 vaccine, which has been approved as a COVID-19 vaccine, induces little neutralizing activity at Week 2 in BALB/cJ mice after a single dose of 0.1 μg or 1.0 μg [21]; however, a 10 μg single dose elicits a substantial anti-pseudovirus-neutralizing titer (IC_50_ GMT = 315) at Week 2, and double this GMT at Week 4, similar to the results of the prime immunization of S2P-NDVLP. Moreover, a single immunization of Luc mRNA-LNP in mice can elicit potent anti-SARS-CoV-2-neutralizing titers (IC_50_, 2 × 10^3^) at Week 9 postimmunization along with very high levels of spike-specific IgG (endpoint, 6 × 10^6^) [57].

In summary, the results provided here indicate S2P-NDVLP to be a promising COVID-19 vaccine candidate. Our current study aimed at comparing S2P-NDVLP against the soluble S2P trimer in terms of titers and neutralizing antibodies after both prime and boost immunizations. Whether the neutralization titers we observed with a single dose of S2P-NDVLP will enable its use as a single-shot vaccine will need further study to determine the duration of humoral and T-cell responses as well as the protective efficacy. The challenges we encountered in this study led us to improve NDVLP purification to obtain VLP immunogens of suitable purity and quality; it will be interesting to see if a single-transcript, mRNA-based S2P-NDVLP vaccine could provide an alternative way to deliver S2P-NDVLP without purification.

## 5. Patents

The authors declare that an intellectual property application has been filed.

## Data Availability

The data generated in this study are contained in this paper and its associated Supplementary Materials.

## Supplementary Materials

The following are available online at https://www.mdpi.com/2076-393X/9/2/73/s1, Figure S1: Purification process of S2P-NDVLP; Figure S2: Quantification of S2P on the surface of S2P-NDVLP; Figure S3: IgG subtype responses.
