# Supplementary material for: Newcastle Disease Virus-Like Particles Displaying Prefusion-Stabilized SARS-CoV-2 Spikes Elicit Potent Neutralizing Responses
Source: Vaccines (Basel). 2021 Jan 21;9(2):73. doi: 10.3390/vaccines9020073 (PMC7912142; doi:10.3390/vaccines9020073)
Supplement: Supplementary file 1 [file vaccines-09-00073-s001.pdf]

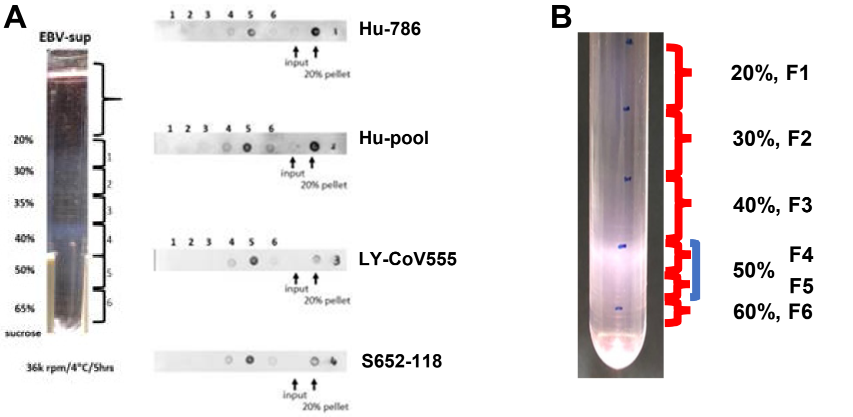

Fraction	OD450
F1	0.1695
F2	0.1762
F3	0.6365
F4	2.1561
F5	2.3842
F6	1.6233
MEDIUM	0.0548
Blank	0.0555

Figure S1. Purification process of S2P-NDVLP. (A) purification of S2P-NDVLP at a small-scale process. Secreted S2P-NDVLP was concentrated from the cleared cell culture supernatant and subjected to a continuous sucrose gradients (20% to 65% sucrose) centrifugation. S2P-NDVLP in the 50% sucrose fraction was verified by immune dot blotting with human anti-SARS-CoV-2 sera. **(B)** purification of S2P-NDVLP at a large-scale process. The white fluffy layer (F4) and light brown layer (F5) in the 50% sucrose fraction were harvested and verified to contain S2P-NDVLP by ELISA with a SARS-CoV-2 neutralizing antibody S652-118 in right panel.

A

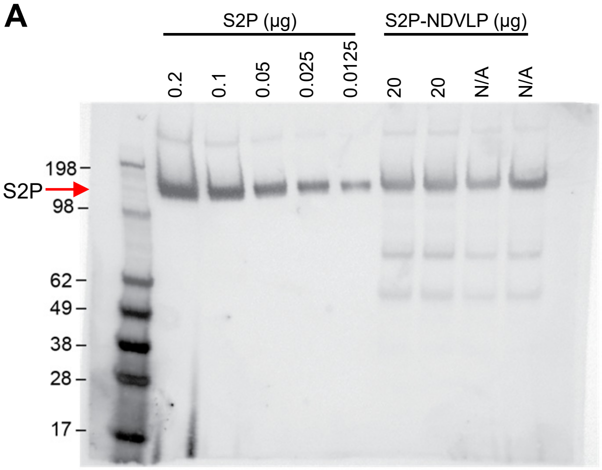

B

	Intensity (AU)	
Protein µg	S2P	S2P-NDVLP
0.2	24679	
0.1	17524	
0.05	9937	
0.025	5060	
0.0125	1239	
20		8737
20		7323

C

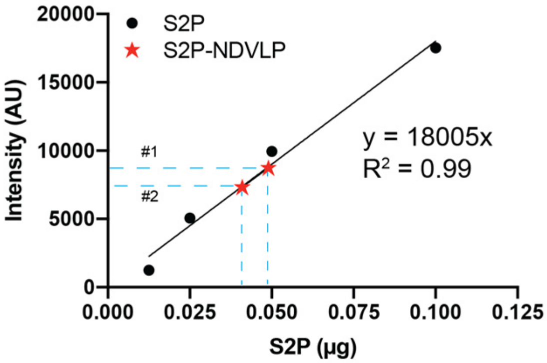

Figure S2. **Quantification of S2P on S2P-NDVLP**. **(A)** Western immunoblot depicts a series of concentrations of soluble S2P protein (0.0125 - 0.2 µg) and samples of S2P-NDVLP. See Materials and Methods for details of the experimental procedure. The last two lanes of S2P-NDVLP were uncertain of the amount loaded and thus were not used for quantitation. **(B)** densitometry readings (intensity) of each band in (A). **(C)** A standard concentration curve *vs.* corresponding densitometry signal of S2P-standards and readings of S2P amount in S2P-NDVLP samples (red asterisks) from the standard curve. One µg of the S2P-NDVLP sample in the quantification contains 0.0025 µg of S2P spike protein. Thus, the amount of S2P on the surface of S2P-NDVLP: 1 µg of S2P-NDVLP = 0.0025 µg of S2P protein.

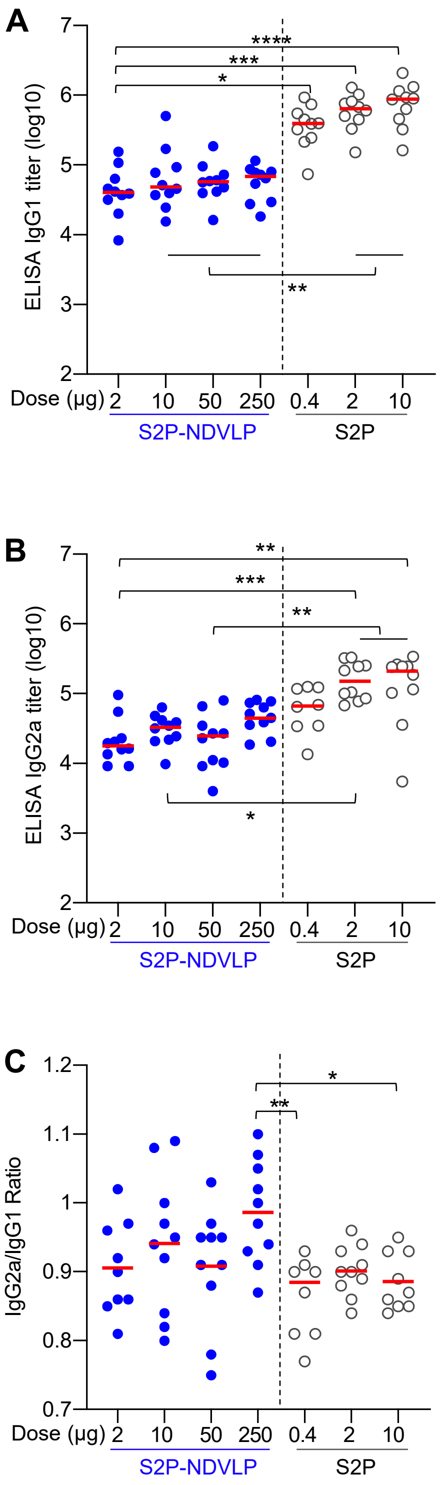

Figure S3. **IgG subclass ratio responses**. BALB/c mice were immunized with S2P-NDVLP (2-250 µg) or soluble trimer S2P (0.4-2 µg) at weeks 0 and 3. Sera from post-boost mice were assessed for ratio of IgG subtypes. **(A)** IgG1 endpoint titers, **(B)** IgG2a endpoint titers, and **(C)** ratio of IgG2a/IgG1 titers for each mouse of the immunization groups. IgG1 is indicative of a Th2 response, and IgG2 of a Th1 response. IgG2a/IgG1 ratios were calculated after taking log10 of the endpoint titers. Both soluble trimer and S2P-NDVLP produced a balanced Th1-Th2 response in all groups immunized with various doses. Each dot represents an individual mouse with the solid red line representing geometric mean titer in (A) and B, and mean IgG2a/IgG1 titer ratio in (C). Open circles are for S2P-immunized groups, and blue filled circles are for S2P-NDVLP-immunized groups. Mice that did not reach endpoint titer were excluded from the ratio analyses. Kruskal-Wallis test was used to test differences between the groups.
